# Prospective analysis of post-COVID-19 hospitalization: Impact on pulmonary function, physical performance and overall functionality

**DOI:** 10.1016/j.clinsp.2026.100938

**Published:** 2026-04-18

**Authors:** Danielle Brancolini de Oliveira, Gabriela Sayuri Ochiai, Elizabeth Mendes da Silva, Camila Machado de Campos, Caroline Gil de Godoy, Erika Christina Gouveia e Silva, Celso Ricardo Fernandes Carvalho, Jose Eduardo Pompeu

**Affiliations:** Department of Physical Therapy, Speech Therapy and Occupational Therapy, Faculdade de Medicina, Universidade de São Paulo, São Paulo, SP, Brazil

**Keywords:** COVID-19, Post-acute COVID-19 syndrome, Respiratory function tests, Physical functional performance

## Abstract

**Background:**

COVID-19 can lead to enduring long-term effects, particularly in severe cases requiring hospitalization. This study contrasts Normal lung Patterns (NP) with Restrictive lung Disease (RD) regarding the evolution of physical performance and functionality post-COVID-19 hospitalization.

**Methods:**

This is a prospective cohort study. Individuals post-COVID-19 were evaluated at 1- and 12-months post-discharge. Data collection included analysis of lung function, physical performance, functionality, and clinical data.

**Results:**

Of the 185 participants initially assessed, 62 attended the final evaluation. At 1 month post-discharge, 57 (92%) had been admitted to the ICU and 47 (76%) exhibited RD. After 12-months, RD decreased to 20 (32%). Both groups showed improved Sit-to-Stand repetitions (p = 0.002). A substantial percentage (28%) of participants with RD experienced functional impairments at one month, with a notable proportion maintaining these limitations after one year (15%).

**Conclusions:**

Almost half of individuals with RD (43%) still exhibited this pulmonary alteration after one year (RD 20/47). Additionally, RD participants demonstrated decreased physical performance at one month (15%), with only a fraction (5%) showing improvement after one year. Finally, these results pointed out the necessity of long-term follow-up regarding the recovery trajectory of post-COVID patients. Furthermore, there is a clear need to offer specific rehabilitation focusing on pulmonary, physical performance and functionality.

## Highlights

• Restrictive lung disease is common after severe COVID-19.
• Lung function improves over one year, but impairment often persists.
• Physical performance improves, especially in restrictive lung disease.
• Functional limitations persist in some patients after one year.
• Long-term follow-up and rehabilitation are essential after COVID-19 hospitalization.

## Introduction

Patients with severe COVID-19 can experience systemic repercussions and may progress to Acute Respiratory Distress Syndrome (ARDS), necessitating Invasive Mechanical Ventilation (IMV). ^1^ Those admitted to the Intensive Care Unit (ICU) are at a higher risk of developing Long COVID, ^2^ which can result in systemic sequelae. ^3^ These sequelae can appear within the first-week post-discharge and persist for 6- to 12-months. ^4^

Notably, respiratory system changes have been identified, including restrictive lung physiology and fibrotic modifications, and they affect at least 10% of post-COVID patients by the third-month post-infection. ^2^^,^^5^^,^^6^ Restrictive lung disease (RD) was observed in 50% of cases three months post-COVID and was the most prevalent pulmonary impairment six months post-discharge.^5^^,^^7^, ^8^, ^9^ RD was present in 98% of patients who required ICU admission, ^5^ correlating directly with disease severity. ^10^ This condition may be developed due to pulmonary fibrotic changes caused by COVID-19, although some studies suggest that pulmonary involvement might reverse after 12-months.^5^^,^^7^^,^^11^, ^12^, ^13^, ^14^

Post-hospitalization, patients often experience impaired physical performance and functionality,^15^^,^^16^ with reduced capability evidenced by difficulty performing the Sit-to-Stand Test (STS) within the first month after discharge. This impact is more pronounced in patients with prolonged hospital stays of more than ten days. ^15^^,^^17^ However, the long-term trajectory of these sequelae remains unclear.

This study compared physical performance and functionality progression between patients with Normal Pulmonary function (NP) and those with Restrictive lung Disease (RD) one and twelve months after COVID-19 hospitalization.

## Materials and methods

### Study cohort

This prospective cohort was approved by the Institutional Ethics Committee of the School of Medicine of the University of Sao Paulo (4.135.985, July 6, 2020), and all participants gave written informed consent to participate in the study. Clinical trial number: not applicable. The present study was conducted and reported in accordance with the STROBE (Strengthening the Reporting of Observational Studies in Epidemiology) Statement.

The study included participants with a history of hospitalization due to COVID-19 at the Hospital das Clinicas of the University of Sao Paulo. Participants were assessed at two-time points: one and 12-months post-hospital discharge. The collection period took place from June 2020 to July 2021.

The exclusion criteria included inability to complete follow-up within 12-months post-discharge, presence of cognitive impairments that hindered comprehension of the tests, and pulmonary function indicative of obstructive lung disease.

### Lung function – spirometry

Lung function was assessed using a portable digital spirometer (“Koko”, SX 1000), administered by a trained professional from the research center. The procedure adhered to guidelines set forth by the American Thoracic Society and European Respiratory Society. ^18^ The analysis included absolute and predicted values for the Brazilian population concerning Forced Vital Capacity (FVC), forced expiratory volume in the First Second (FEV1), and the FEV1/FVC ratio. An FVC value less than 80% of the predicted value was classified as indicative of Restrictive lung Disease (RD) .^19^^,^^20^ The exclusion of obstructive disorders was deliberate in order to isolate the restrictive effects directly attributable to COVID-19, minimizing confounding by pre-existing conditions.

### Physical performance – STS1′

The sit-to-stand test was conducted using a standard-height chair (46 cm) without armrests, with participants instructed to cross their arms and place their hands on their shoulders.^21^^,^^22^ The MCID established for patients with Chronic Obstructive Pulmonary Disease (COPD) was adopted to assess improvements between two testing intervals.^23^ An increase of three repetitions in STS1 is considered a clinically significant improvement.^23^

### Functionality – Barthel index (BI)

The same physiotherapist administered the functionality scale to ensure consistency. The Barthel Index (BI) was utilized to evaluate functional capacity and independence in activities of daily living.^24^^,^^25^ The cumulative score of the assessed functions was used to determine the patient's degree of dependency. A total score below 85-points is indicative of functional impairment.^26^^,^^27^

### Statistical analyses

Statistical analysis commenced with the Shapiro-Wilk test to assess data normality. The unpaired Student's *t*-test and Mann-Whitney test (depending on the distribution) were used, and the Chi-Square test was used for the nominal variables. A comparative analysis of baseline characteristics (1st-month) was performed between participants who completed the study (n = 62) and those who dropped out (n = 123) to verify the attrition rate. Lung function at 1- and 12-months post-discharge was compared using the Chi-Square test (χ²) for categorical variables. Repeated measures ANOVA was employed to evaluate changes in continuous variables across the two time periods. Data entry and tabulation were conducted using the Research Electronic Data Capture Management Platform (REDCap), while statistical processing was performed with JASP software. A p-value of less than 0.05 was considered to indicate statistical significance.

## Results

**Fig. 1:**
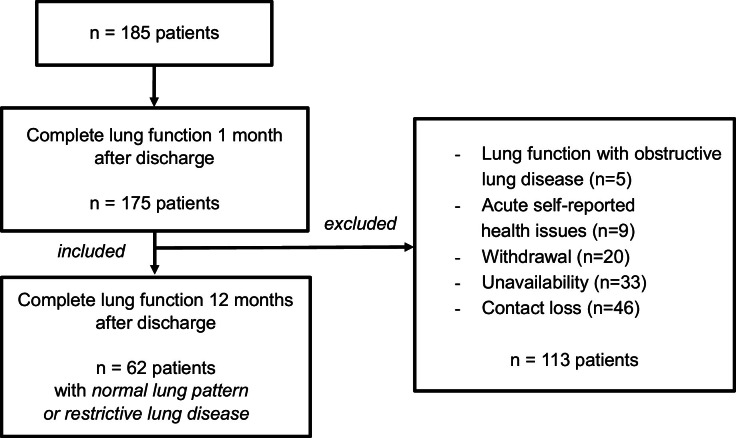
Flowchart for grouping the patients.

A total of 185 individuals participated in the initial evaluation conducted 30 to 45 days post-discharge. In the follow-up evaluation undertaken 12-months after discharge, 62 individuals provided complete pulmonary function test data. Participants exhibiting obstructive lung disease in their pulmonary function tests were excluded from the study (refer to Fig. 1).

### Sociodemographic and clinical characteristics of participants at baseline (1st-month post-discharge)

To verify the attrition rate, a comparative analysis of baseline characteristics (1st-month) was performed between participants who completed the study (n = 62) and those who dropped out (n = 123). Of the 123 participants, 68 (57%) were men, with a mean age of 58 (12) years (Table 1). The median length of stay was 17 (10‒30) days; 94 (79%) were admitted to the ICU, and the median time in the ICU was 10 (6‒19) days; 59 (51%) patients required IMV.

**Table 1:** Baseline characteristics between dropouts and retained participants.

Characteristics	Total including dropouts (n = 123)	Retained participants (n = 62)	p
Age, year m (SD)	58 (13)	58 (12)	0.842
Sex n (%) Male Female	68 (57) 52 (43)	32 (52) 30 (48)	0.658
Length of stay, days median (IQR)	17 (10–30)	26 (17–35)	0.653
ICU admission n (%)	94 (79)	57 (92)	0.026
Length of stay in ICU, days median (IQR)	10 (6–19)	14 (7–22)	0.519
IMV needed n (%)	59 (51)	46 (74)	0.017

n, Absolute number; NP, Normal lung Pattern; RD, Restrictive lung Disease; m, Mean; SD, Standard Deviation; IQR, Interquartile Range; ICU, Intensive Care Unit; IMV, Invasive Mechanical Ventilation.

There was no statistically significant difference between the groups with respect to age, sex, length of hospital stay and length of ICU stay. There was a significant difference between admission in ICU (p = 0.026) and IMV needed (p = 0.017).

Of the 62 participants evaluated at both moments, 32 (52%) were men, with a mean age of 58 (12) years (Table 2). The median length of stay was 26 (17‒35) days; 57 (92%) were admitted to the ICU, and the median time in the ICU was 14 (7‒22) days; 46 (74%) of the total sample required IMV, and the median time was 5 (0.3‒10) days; 57 (92%) patients required O_2_ on the first day of hospitalization. There was a prevalence of 25 (40%) individuals who presented pulmonary impairment between 50%‒100% on CT scan (Table 2). There was no significant difference in clinical and sociodemographic characteristics between participants who dropped out of the study and those who remained in the final analysis, demonstrating that there was no attrition.

**Table 2:** Sociodemographic and clinical characteristics of the study population and the NP and RD groups.

Characteristics	Total (n = 62)	NP (n = 15)	RD (n = 47)
Age, year m (SD)	58 (12)	59 (14)	58 (11)
Sex n (%) Male Female	32 (52) 30 (48)	9 (60) 6 (40)	23 (49) 24 (51)
Length of stay, days median (IQR)	26 (17–35)	19 (16–25)	29 (21–40)
ICU admission n (%)	57 (92)	13 (87)	44 (94)
Length of stay in ICU, days median (IQR)	14 (7–22)	13 (6–19)	14 (7–25)
IMV needed n (%)	46 (74)	7 (47)	39 (83)
Time on IMV, days median (IQR)	5 (0.3–10)	0 (0–6)	7 (3–10)
O_2_ needed on the first day n (%)	57 (92)	11 (73)	46 (98)
O_2_ length, days median (IQR)	4 (2–7)	2 (1–5)	5 (2–8)
Oxygen therapy n (%) Nasal cannula (1–5L/min) Non-rebreather (5–15L/min)	38 (61) 19 (31)	6 (40) 5 (33)	32 (68) 14 (30)
NIV n (%)	10 (16)	4 (27)	6 (13)
VAD n (%)	41 (66)	5 (33)	36 (77)
Home O_2_ therapy n (%)	3 (5)	0 (0)	3 (6)
Pulmonary impairment by CT scan n (%) Up to 50% 50% 50–100%	21 (33) 16 (26) 25 (40)	9 (60) 4 (27) 2 (13)	12 (26) 12 (26) 23 (49)

n, Absolute number; NP, Normal lung Pattern; RD, Restrictive lung Disease; m, Mean; SD, Standard Deviation; IQR, Interquartile Range; ICU, Intensive Care Unit; IMV, Invasive Mechanical Ventilation; O_2_, Oxygen; NCO_2_, Nasal Cannula; NRB, Non-Rebreather; NIV, Non-Invasive Ventilation; VAD, Vasoactive Drug; CT scan, Computed Tomography.

### Lung function in the 1st and 12th months

Table 3 displays the absolute and predicted values from the pulmonary function tests for both the NP and RD groups.

**Table 3:** Patients’ lung function in the 1st and 12th-months in NP and RD groups.

			Mean Difference (CI 95%)		Assessment		Group		Interaction	
Variables m (SD)	1st month	12th month	between 1st and 12th months	between NP and RD	p	ω^2^	p	ω^2^	p	ω^2^
FVC predicted (%)										
NP RD	91.0 (8.1) 65.0 (12.6)	100.0 (9.8) 79.0 (15.3)	−11.5 (−15.3;−7.8)^a^	23.4 (16.6;30.2)^a^	<0.001	0.12	<0.001	0.28	0.371	0.00
FEV1 predicted (%)										
NP RD	97.0 (18.5) 69.0 (13.7)	104.0 (13.7) 88.0 (49)	−13.0 (−25.4;−0.6)^c^	21.8 (6.8;37.0)^b^	0.040	0.02	0.005	0.06	0.336	0.00
FEV1/FVC predicted (%)										
NP RD	107.0 (10.2) 110.0 (8.7)	104.0 (8.8) 106.0 (7.1)	3.2 (1.0;5.4)^b^	−2.0 (−6.4;2.4)^d^	0.005	0.02	0.372	0.00	0.498	0.00

ANOVA repeated measures.

n, Absolute number; NP, Normal lung Pattern; RD, Restrictive lung Disease; m, Mean; SD, Standard Deviation; %, Percentage of predicted value; FVC, Forced Vital Capacity; FEV1, Forced Expired expiratory Volume in the 1st second.

^a^Bonferroni post hoc test (p < 0.001***); ^b^ Bonferroni post hoc test (p = 0.002**); ^c^ Bonferroni post hoc test (p = 0.040*); ^d^ Bonferroni post hoc test (p = 0.261). ω^2^, omega squared (trivial < 0.01; small 0.01; medium 0.06; large 0.14).

#### FVC predicted value (%)

Regarding the FVC analysis, there was an evaluation effect (*F* = 38.2, p < 0.001, ω^2^ = 0.12) and a group effect (*F* = 47.5, p < 0.001, ω^2^ = 0.28) that was a large effect based on omega squared, but no interaction effect (*F* = 0.8, p = 0.371, ω^2^ = 0.00). Both groups showed an increase in the predicted FVC value over time at 12-months post-discharge (mean difference = −11.5, p < 0.001), and this increase was more significant in RD (mean difference = 23.0, p < 0.001) (Table 3).

#### FEV_1_ predicted value (%)

There was an evaluation effect (*F* = 4.4, p = 0.040, ω^2^ = 0.02) and a group effect (*F* = 8.4, p = 0.005, ω^2^ = 0.06), but no interaction effect (*F* = 0.9, p = 0.336, ω^2^ = 0.00). Both groups showed an increase in the predicted value of FEV_1_ at 12-months post-discharge (mean difference = −13.0, p = 0.040), and this increase was more significant in RD (mean difference = 21.8, p = 0.005) (Table 3).

#### FEV_1_/FVC ratio predicted value (%)

There was an evaluation effect (*F* = 8.5, p = 0.005, ω^2^ = 0.02), but no group effect (*F* = 0.8, p = 0.372, ω^2^ = 0.00) or interaction effect (*F* = 0.5, p = 0.498, ω^2^ = 0.00). Both groups showed an increased FEV_1_/FVC ratio predicted value at 12-months post-discharge (mean difference = 3.2, p = 0.005) (Table 3).

**Fig. 2:**
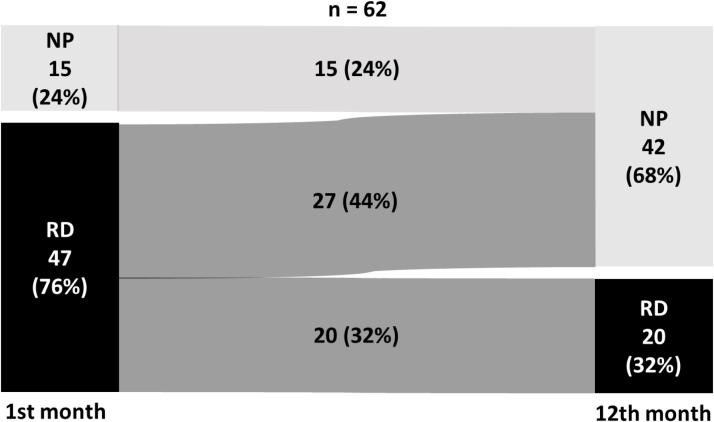
Patient's lung pattern classified as NP and RD in the 1st- and 12th-month. n, Absolute Number; NP, Normal lung Pattern; RD, Restrictive lung Disease.

Fig. 2 shows that 15 (24%) individuals presented NP in the 1st-month and 42 (68%) in the 12th-month, while 47 (76%) individuals presented RD in the 1st-month and 20 (32%) in the 12th-month. Of those who presented RD in the 1st-month, 27 (44%) started to present NP in the 12th-month (Fig. 2). The χ^2^ Test showed a significant difference (p = 0.002) between the groups between the 1st- and 12th-months post-discharge (Fisher's exact test, p = 0.001) (Fig. 2).

### Physical performance and functionality

Table 4 presents the results of STS1 and BI divided into NP and RD groups in the 1st- and 12th-months after discharge.

**Table 4:** Patients’ physical performance and functionality in the 1st- and 12th-months in NP and RD groups.

			Mean Difference (95% CI)		Assessment		Group		Interaction	
Variables	1st month	12th month	Between 1st and 12th months	Between NP and RD	p	ω^2^	p	ω^2^	p	ω^2^
**STS1’**										
NP	18.8 (5.4)	19.5 (6.3)	−2.2 (−3.5; −0.9)^a^	1.8 (−0.8; 4.5)^b^	0.002	0.04	0.175	0.02	0.022	0.01
RD	15.5 (4.3)	19.1 (4.4)								
**BI**										
NP	95.3 (7.8)	96.0 (7.6)	−2.6 (−6.5; 1.3)^b^	3.2 (−2.7; 9.1)^b^	0.194	0.00	0.278	0.00	0.948	0.00
RD	90.2 (14.6)	94.6 (10.9)								

ANOVA repeated measures.

m, Mean; SD, Standard Deviation; NP, Normal lung Pattern; RD, Restrictive lung Disease; STS’, 1-minute Sit and Stand Test; BI, Barthel Index.

^a^Bonferroni post hoc test (p = 0.002**); ^b^Bonferroni post hoc test (p = 0.261). ω^2^, omega squared (trivial < 0.01; small 0.01; medium 0.06; large 0.14).

### Physical performance - STS1′

There was an evaluation effect (*F* = 11.2, p < 0.002, ω^2^ = 0.04) and an interaction effect (*F* = 5.5, p = 0.022, ω^2^ = 0.02), but no group effect (*F* = 1.9, p = 0.175, ω^2^ = 0.01). Both groups showed an increase in the number of TSL1 repetitions at 12-months post-discharge (mean difference = −2.2, p < 0.002), with an interaction between groups (mean difference = 1.8, p = 0.022) (Table 4).

### Functionality - BI

There was no evaluation effect (*F* = 1.7, p = 0.194, ω^2^ = 0.00), group effect (*F* = 1.2, p = 0.278, ω^2^ = 0.00), or interaction effect (*F* = 0.9, p = 0.334, ω^2^ = 0.00). Both groups showed an increase in the total BI score over time, at 12-months post-discharge, without significance (Table 4).

## Discussion

The present study investigated lung function impairment in individuals hospitalized for COVID-19, examining outcomes at 1- and 12-months post-discharge. Due to the emergency nature of the study, it was not possible to assess pre-COVID-19 pulmonary and functional levels. The authors focused on comparing those with Restrictive lung Disease (RD) against individuals with Normal Pulmonary function (NP) in terms of the evolution of physical performance and functionality over time. Initially, most patients (76%) exhibited RD one month after discharge, with both Forced Vital Capacity (FVC) and Forced Expiratory Volume in one second (FEV1) below predicted values. Notably, only 44% of individuals with RD transitioned to NP, but almost half (43%) of individuals who initially presented with DR still had the pulmonary alteration after one year (persistence in 20 of the 47 initial patients with DR), indicating that a portion of participants did not improve their pulmonary function. The RD group initially displayed worse physical performance and lower functionality than the NP group. However, after 12-months, both groups showed similar physical and functional performance.

The present findings underscore the high prevalence (76%) of pulmonary involvement, particularly RD, in critically ill patients with COVID-19 at one-month post-discharge. This result aligns with existing literature identifying the lungs as highly susceptible to COVID-19 damage.^14^^,^^28^ RD is the predominant lung impairment due to pulmonary fibrosis commonly found in severe cases.^5^^,^^7^, ^8^, ^9^ Patients with severe COVID-19 frequently presented pneumonia, which contributed to compromised lung function, persisting three months into recovery.^7^ Post-discharge studies further emphasize the significance of fibrotic lung changes observed on chest CT scans in elucidating restrictive pathology, with fibrosis detected in 10% of patients exhibiting persistent symptoms three months post-infection.^5^ These findings reinforce clinical guidelines from the British Thoracic Society, which recommend monitoring lung function in severe COVID-19 pneumonia cases for up to 12-weeks following discharge.^6^

Longitudinal studies on COVID-19 survivors suggest that pulmonary sequelae can extend 6-to 12-months after infection.^4^ RD remains the most common pulmonary issue six months post-infection and persists in those requiring ICU care in 98% of instances.^5^

The present study demonstrated that after 12-months, 32% of participants continued to exhibit RD. Improvements in FVC and FEV1 were observed in both groups, particularly pronounced within the RD group, which started with lower baseline function. Despite this progress, the RD group did not achieve 100% of predicted FVC values even after 12-months, maintaining only 79% of the expected level. This result indicates that long-term lung impairment may persist in critically ill patients after COVID-19.

These observations may be attributed to the severity of the disease, as patients facing extended hospitalizations and requiring intensive care exhibit more substantial and enduring lung damage. There was a prevalence of 25 (40%) individuals with pulmonary impairment between 50%‒100% on CT scan, with 23 (49%) in the RD group. These findings corroborate results previously obtained through lung function tests and CT scans.^5^^,^^7^, ^8^, ^9^^,^^29^^,^^30^ This trend is similar to observations from a prospective cohort study involving 94 survivors of SARS-CoV-1, which reported persistent lung function impairments in about a third of patients after one year.^12^ These survivors also experienced significantly poorer health outcomes than the general population.^12^

Similarities with the 2019 Coronavirus include observed diffusion capacity reductions and interstitial fibrosis presence^3^ characterized by the thickening and stiffening of lung tissues due to the replacement of normal tissue with scar tissue (collagen), which hinders gas exchange and breathing and leads to deconditioning. 3,5,7, 8, 9] These conditions can persist for 6-months to 15-years after infection.^3^ Among non-critical COVID-19 patients, spirometry did not reveal significant differences in lung function before and after one year of the infection^31^, contrasting this cohort, which consists of severely ill patients (92% ICU, 74% IMV). However, underlying conditions like interstitial lung disease and cystic fibrosis were associated with decreased lung function following infection.^31^ Few studies demonstrate complete recovery of lung function after 1 year, highlighting the discrepancies in relation to disease severity and access to rehabilitation.^12^^,^^31^

Regarding physical performance, the RD group demonstrated lower capabilities in the first month post-discharge than the NP group, as evidenced by fewer repetitions on the sit-to-stand test. Previous studies evaluating COVID-19 patients have similarly noted impairments in physical performance at discharge^17^ and one month afterward.^15^ This decline in physical performance has been associated with reduced oxygen saturation during exertion and increased dyspnea, especially in patients who experienced prolonged hospital stays of more than 10-days.^15^^,^^32^ Consequently, pulmonary impairment directly impacts physical performance, underscoring the need for comprehensive, multi-faceted interventions that address both motor and functional aspects tailored to individual patient needs.

After 12-months, both groups demonstrated an increase in sit-to-stand test repetitions, with the RD group showing a clinically significant improvement based on 3 repetitions by MCID^33^, bringing their results closer to those of the NP group. Although the RD group showed significant improvement (p = 0.022), this average improvement may not yet have reached full clinical relevance at 12-months, although the trend is positive. This finding suggests that patients with restrictive pulmonary impairment can achieve notable improvements in physical performance over time. Existing studies indicate that most survivors experience substantial pulmonary and physical recovery from the disease.^5^^,^^13^^,^^16^^,^^34^

Regarding functionality, the RD group recorded lower scores on the Barthel Index (BI) during the first-month post-discharge. However, both groups approached their maximum score at each evaluation point. A significant proportion (28%) of participants in the RD group exhibited functional impairments one month after discharge, with a notable percentage (15%) persisting in these limitations after one year. These findings contrast with other studies that reported functional impairments at one-month post-discharge using the BI.^35^ Research indicates that hospitalization for COVID-19 negatively impacts functional capacity, particularly among older adults, with sarcopenia and frailty identified as contributing factors.^35^ Additionally, age, hyperglycemia, and the time required to start mobilizing patients from their beds have been independently associated with functional impairment following ICU admission.^36^ Previous studies have highlighted significant deficits in mobility activities at discharge^36^, as well as impairments in feeding^4^, bathing, and transfers^16^, providing critical insights for healthcare professionals to develop targeted interventions aimed at alleviating these effects. The cohort was highly dependent on the ICU (92%) with an average time of 14-days, which reinforces the focus on critically ill patients. Future studies are needed to correlate these outcomes with specific ICU interventions.

### Limitations and strengths of the study

It was not feasible to assess participants' lung function, physical performance, and functionality prior to COVID-19 hospitalization, which precluded pre and post-hospitalization comparisons of these parameters. Patients with obstructive lung disease were excluded to eliminate potential confounding from pre-existing conditions unrelated to COVID-19, limiting comparisons to individuals with RD versus obstructive disorders.

The significant differences in ICU admission and IMV need between completers and dropouts suggest a potential generalization bias. Those who remained in the study may not fully represent the initial cohort's clinical severity. Despite this, no significant differences were observed in hospital and ICU lengths of stay between the two groups.

The strengths of this study include its prospective cohort design, which provides a comprehensive evaluation of clinical, physical, and functional outcomes over one year. These results offer valuable insights for public health initiatives, informing the development of treatment approaches for patients hospitalized due to COVID-19.

Given the persistent sequelae observed as early as the first-month post-discharge, ongoing follow-up for this population is crucial. The potential to accelerate recovery through rehabilitative interventions represents a valuable opportunity to improve quality of life and address socioeconomic implications.

### Clinical implications and recommendations for future research

Considering the significant impact of COVID-19 on public health, quality of life, lung function, physical performance, and functionality, ongoing monitoring and evaluation of affected individuals are crucial. It is essential to implement targeted strategies at the time of discharge and shortly after that to effectively mitigate both the immediate effects of the disease and the complications associated with prolonged hospitalization. Such proactive measures are vital for optimizing recovery outcomes and enhancing the overall well-being of this population.

## Conclusions

Restrictive lung disease was identified in 76% of participants one month post-COVID-19 hospitalization and in 32% at the 12-month. Individuals with RD initially displayed lower physical performance and functional capacity than individuals with normal pulmonary function. However, participants with RD exhibited more significant improvements in physical and functional outcomes over time, whereas the NP group maintained relatively high-performance levels from the outset. These findings illustrate the potential for recovery in individuals with RD and underscore the importance of continued monitoring and rehabilitation efforts in this population.

## Declarations

### Ethics approval and consent to participate

This prospective cohort was approved by the Institutional Ethics Committee of the School of Medicine of the University of Sao Paulo (4.135.985, July 6, 2020), and all participants gave written informed consent to participate in the study.

### Consent for publication

Not applicable.

### Data availability statement

The data presented in this study are available from the corresponding author upon reasonable request.

## Authors’ contributions

**Danielle Brancolini de Oliveira** was the chief investigator, responsible for the data analysis and drafting the original manuscript. **Caroline Gil de Godoy, Elizabeth Mendes da Silva, Camila Machado de Campos** and **Gabriela Sayuri Ochiai** made substantial contributions to the conception, investigation and data curation of the work. **Erika C. G. Silva** and **Celso R. F. Carvalho** substantially contributed to the revision of the manuscript drafts. **Jose Eduardo Pompeu** was responsible for the organization and coordination of the study. All authors have approved the submitted version of the manuscript and agree to be accountable for any part of the work.

## Funding

This study was funded by the National Council for Scientific and Technological Development (CNPq), process n° 311070/2020-5, and by the State of Sao Paulo Research Foundation (FAPESP), process n° 2018/19618-8.

## Declaration of competing interest

The authors declare no conflicts of interest.

## References

[1] Izzaty R.E., Astuti B., Cholimah N. (2020). Characteristics of SARS-CoV-2 and COVID-19. Nat Rev Microbiol.

[2] Nalbandian A., Sehgal K., Gupta A., Madhavan M.V., Mcgroder C., Stevens J.S. (2021). Post-acute COVID-19 syndrome. Nat Med.

[3] Piotrowicz K., Gasowski J., Michel J.P., Veronese N. (2021). Post-COVID-19 acute sarcopenia: physiopathology and management. Aging Clin Exp Res.

[4] Wiertz C., Vints W., Maas G., Rasquin S., Horn Y., Dremmen M. (2021). COVID-19: patient characteristics in the first phase of postintensive care rehabilitation. Arch Rehabil Res Clin Transl.

[5] Bretas D.C., Leite A.S., Mancuzo E.V., Prata T.A., Andrade B.H., Oliveira J.G.F. (2022). Lung function six months after severe COVID-19: does time, in fact, heal all wounds?. Brazilian J Infect Dis.

[6] British Thoracic Society. British Thoracic Society guidance on Respiratory follow up of patients with a clinico-radiological diagnosis of COVID-19 pneumonia. Br Thorac Soc guid respir follow up patients with a clin diagnosis COVID-19 Pneumonia 2020;1:2.

[7] Salem A.M., Al Khathlan N., Alharbi A.F., Alghamdi T., Alduilej S., Alghamdi M. (2021). The long-term impact of covid-19 pneumonia on the pulmonary function of survivors. Int J Gen Med.

[8] Jimeno-Almazan A., Pallares J.G., Buendia-Romero A., Martinez-Cava A., Franco-Lopez F., Sanchez-Alcaraz B.J. (2021). Post-COVID-19 syndrome and the potential benefits of exercise. Int J Environ Res Public Health.

[9] Torres-Castro L., Vasconcello-Castillo X., Alsina-Restoy L., Solis-Navarro F., Burgosc H., Puppoa J.V. (2021). Respiratory function in patients post-infection by COVID-19. J Pulmonol.

[10] George P.M., Barratt S.L., Condliffe R., Desai S.R., Devaraj A., Forrest I. (2020). Respiratory follow-up of patients with COVID-19 pneumonia. Thorax.

[11] Blanco J., Navarro F., Sanjoaquin I., Arnaiz F., Revillas D., Bernal E. (2021). Pulmonary long-term consequences of COVID-19 infections after hospital discharge. Clin Microbiol Infect J.

[12] Kian-Chung O., Alan N., Lawrence L.S., Gregory K., Seow-Khee K., Khee-Shing L. (2005). 1-Year pulmonary function and health status in survivors of severe acute Respiratory syndrome. Chest.

[13] Wu X., Liu X., Zhou Y., Yu H., Li R., Zhan Q. (2021). 3-month, 6-month, 9-month, and 12-month respiratory outcomes in patients following COVID-19-related hospitalisation: a prospective study. Lancet Respir Med.

[14] Hui D.S., Wong K.T., Ko F.W., Tam L.S., Chan D.P., Woo J. (2005). The 1-year impact of severe acute respiratory syndrome on pulmonary function, exercise capacity, and quality of life in a cohort of survivors. Chest.

[15] Nunez-Cortes R., Rivera-lillo G., Rodrigo N., Arias-Campoverde M., Soto-Garc D., Garcia Palomera R. (2021).

[16] Belli S., Balbi B., Prince I., Cattaneo D., Masocco F., Zaccaria S. (2020). Low physical functioning and impaired performance of activities of daily life in COVID-19 patients who survived the hospitalisation. Eur Respir J.

[17] Paneroni M., Simonelli C., Saleri M., Bertacchini L., Venturelli M., Troosters T. (2021). Muscle strength and physical performance in patients without previous disabilities recovering from COVID-19 pneumonia. Am J Phys Med Rehabil.

[18] Graham B.L., Steenbruggen I., Barjaktarevic I.Z., Cooper B.G., Hall G.L., Hallstrand T.S. (2019). Standardization of spirometry 2019 update an official American Thoracic Society and European Respiratory Society technical statement. Am J Respir Crit Care Med.

[19] Pereira C.A.C., Sato T., Rodrigues S.C (2007). New reference values for forced spirometry in white adults in Brazil. J Bras Pneumol.

[20] Ranu H., Wilde M., Madden B. (2011). Pulmonary function tests. Ulster Med J.

[21] Reychler G., Boucard E., Peran L., Pichon R., Ber-Moy C., Ouksel H. (2018). One minute sit-to-stand test is an alternative to 6MWT to measure functional exercise performance in COPD patients. Clin Respir J.

[22] Bohannon R.W., Crouch R. (2019). 1-Minute sit-to-stand test: systematic review of procedures, performance and clinimetric properties. J Cardiopulm Rehabil Prev.

[23] Rugila D.F., Bisca G.W., Corso S.D., Furlanetto K.C. (2021). Interpretação da capacidade funcional de membros inferiores valores normativos para a prática clínica. Vol. 2, Programa De Atualização Profisio - Fisioterapia Cardiovascular E Respiratória.

[24] Shan S., Vancley F., Cooper B. (1989). Improving the sensitivity of the barthel index for stroke rehabilitation. J Clin Epidemiol.

[25] Minosso J.S.M., Amendola F., Alvarenga M.R.M., Oliveira M.A.C. (2010). Validação, no Brasil, do Índice de Barthel em idosos atendidos em ambulatórios. Acta Paul Enferm.

[26] Katano S., Yano T., Ohori K., Kouzu H., Nagaoka R., Honma S. (2022). Barthel Index score predicts mortality in elderly heart failure. Circ J.

[27] Reis N.F., Figueiredo F.C.X.S., Biscaro R.R.M., Lunardelli E.B., Maurici R. (2022). Psychometric properties of the Barthel Index used At intensive care unit discharge. Am J Crit Care.

[28] Martillo M.A., Dangayach N.S., Tabacof L., Spielman L.A., Dams-O'connor K., Chan C.C. (2021). Postintensive care syndrome in survivors of critical illness related to coronavirus disease 2019: cohort study from a New York City Critical Care Recovery clinic. Crit Care Med.

[29] Chan K.S., Zheng J.P., Mok Y.W., Li Y.M., Liu Y.N., Chu C.M. (2003). SARS: prognosis, outcome and sequelae. Respirology.

[30] Latronico N., Peli E., Calza S., Rodella F., Novelli M.P., Cella A. (2021). Physical, cognitive and mental health outcomes in 1-year survivors of COVID-19-associated ARDS. Thorax.

[31] Lewis K.L., Helgeson S.A., Tatari M.M., Mallea J.M., Baig H.Z., Patel N.M. (2021). COVID-19 and the effects on pulmonary function following infection: a retrospective analysis. eClinicalMedicine.

[32] Alonso A.C., Silva-Santos P.R., Quintana M.S.L., Silva V.C., Brech G.C., Barbosa L.G. (2021). Physical and pulmonary capacities of individuals with severe coronavirus disease after hospital discharge: a preliminary cross-sectional study based on cluster analysis. Clinics.

[33] Furlanetto K.C., Correia N.S., Mesquita R., Morita A.A., Amaral D.P., Mont’Alverne D.G.B. (2022). Reference values for 7 different protocols of simple functional tests: a multicenter study. Arch Phys Med Rehabil.

[34] Moreno-pérez O., Merino E., Leon-Ramirez J., Andres M., Manuel J., Arenas-Jiménez J. (2021). Post-acute COVID-19 syndrome. Incidence and risk factors: a Mediterranean cohort study. J Infect J.

[35] Ochiai G.S., Godoy C.G., Silva E.C.G., Oliveira D.B., Silva E.M., Viana O.C. (2023). Functional impact on adults and older people after hospitalization by COVID-19. Physiother Res Int.

[36] Schujmann D.S., Lunardi A.C., Neri C.P., Pompeu J.E., Annoni R., Miura M.C. (2022). Functional recovery groups in critically ill COVID-19 patients and their associated factors: from ICU to hospital discharge∗. Crit Care Med.

